# Cross-sectional study on the weight and length of infants in the interior of the State of São Paulo, Brazil: associations with sociodemographic variables and breastfeeding

**DOI:** 10.1590/S1516-31802009000400004

**Published:** 2009-12-08

**Authors:** Julia Laura Delbue Bernardi, Regina Esteves Jordão, Antônio de Azevedo Barros

**Affiliations:** 1 PhD. Nutritionist in the Department of Pediatrics, Faculdade de Ciências Médicas da Universidade Estadual de Campinas (FCM/Unicamp), Campinas, São Paulo, Brazil.; 2 MSc. Nutritionist in the Department of Pediatrics, Faculdade de Ciências Médicas da Universidade Estadual de Campinas (FCM/Unicamp), Campinas, São Paulo, Brazil.; 3 PhD. Associate professor in the Department of Pediatrics, Faculdade de Ciências Médicas da Universidade Estadual de Campinas (FCM/Unicamp), Campinas, São Paulo, Brazil.

**Keywords:** Growth, Body weight, Body height, Breast feeding, Educational status, Crescimento, Peso corporal, Estatura, Aleitamento materno, Escolaridade

## Abstract

**CONTEXT AND OBJECTIVE:**

Increasing obesity is starting to occur among Brazilians. The aim of this study was to investigate the weight and length of children under two years of age in relation to sociodemographic variables and according to whether they were breastfed.

**DESIGN AND SETTING:**

Cross-sectional randomized study conducted in 2004-2005, based on the declaration of live births (SINASC) in Campinas, Brazil.

**METHODS:**

2,857 mothers of newborns were interviewed and answered a questionnaire seeking socioeconomic and breastfeeding information. The newborns’ weights and lengths were measured at the end of the interviews and the body mass index was calculated. Percentiles (< 15 and > 85) and Z-scores (< -1 and > +1) were used for classification based on the new growth charts recommended by WHO (2006). The log-rank test, multiple linear regression and binomial test (Z) were used. The statistical significance level used was 5%.

**RESULTS:**

The predominant social level was class C. The median for exclusive breastfeeding was 90 days; 61.25% of the children were between P15 and P85 for body mass index and 61.12% for length, respectively. Children whose mothers studied for nine to eleven years and children whose mothers were unemployed presented lower weight. Children whose mothers worked in health-related professions presented lower length when correlated with breastfeeding.

**CONCLUSION:**

The breastfeeding, maternal schooling and maternal occupation levels had an influence on nutrition status and indicated that obesity is occurring in early childhood among the infants living in the municipality.

## RESUMO

**CONTEXTO E OBJETIVO:**

O aumento da obesidade começa a acontecer entre os brasileiros. O objetivo do estudo foi verificar o peso e a altura associados a variáveis sócio-demográficas de crianças menores de dois anos, de acordo com o aleitamento materno.

**TIPO DE ESTUDO E LOCAL:**

Estudo transversal, aleatório, realizado entre 2004 e 2005, com base na Declaração de Nascidos Vivos (SINASC) de Campinas, São Paulo.

**MÉTODOS:**

Mães de 2.857 crianças foram entrevistadas respondendo a um questionário com informações sócio-demográficas e de amamentação. Ao final, foram aferidos peso e comprimento dos lactentes e calculado o Índice de Massa Corporal. Os pontos de percentis (< 15 e > 85) e de escore-Z (< -1 e > +1) foram utilizados para classificação, com base nos novos gráficos de crescimento recomendados pela Organização Mundial de Saúde 2006. Foi utilizado o teste de log-rank, a análise de regressão linear e o teste binomial (Z). O nível de significância estatística adotado foi de 5%.

**RESULTADOS:**

O nível social predominante foi classe C. A mediana do aleitamento materno exclusivo foi de 90 dias. 61,25% das crianças estavam entre os percentis P15-P85 para índice de massa corporal e 61,12% para comprimento, respectivamente. Nas crianças cuja mãe estudou entre 9 e 11 anos o peso foi menor, bem como para os filhos de mães desempregadas. Filhos de mães com profissões na área da saúde apresentaram menor comprimento, quando correlacionados ao aleitamento.

**CONCLUSÃO:**

Os níveis encontrados para aleitamento, escolaridade e profissão maternas exerceram influência sobre o estado de nutrição, indicando que a obesidade está acontecendo precocemente na infância nos lactentes do município.

## INTRODUCTION

Growth is a complex process that begins with conception, and its purpose is to enable evolution to maturity. By the time that human beings reach maturity, many structural modifications are evident. Growth is characterized by increases in total body size or in any of its parts, with associations between hyperplasia and hypertrophy. Development means acquisition of functions and abilities and it has been associated with cell differentiation and maturation of different organs and systems.^1^^,^^2^

If the general conditions are suitable, growth and development will sequentially interact with each other until maturity is reached. The intrauterine phase and the first year of life are the most critical periods, during which the intensity of these parameters undergoes changes.^1^^,^^2^ However, any reference to infant malnutrition should take into account that it is a public health problem in developing countries, given that 38.1% of children under five years of age are estimated to present abnormal length/height and 31% of them, abnormal weight, in these countries.^1^^,^^2^ Non-exclusive breastfeeding over the first six months of life, together with the family’s socioeconomic level, and subsequent inadequate introduction of complementary foods into the diet, may affect the development of dietary habits and growth.^3^^,^^4^ In the specific case of the initial years of life, weight and length measurements are important indicators of growth, in addition to being recommendable in studies aimed at assessing the nutritional status of a population.^5^^,^^6^^,^^7^^,^^8^

In 2000, the Brazilian Ministry of Health (DATASUS)^5^ found that the prevalence of exclusive breastfeeding in Brazil was 53.1% for one month, 21.6% for between three and four months and 9.7% for between five and six months. In the city of São Paulo, these rates were 36.2%, 15.1% and 7.6%, respectively. Low schooling level, young age, primiparity, early maternity and lack of knowledge about breastfeeding practices among the mothers were considered to be risk factors for weaning.

Anthropometric data need to be gathered periodically in order to monitor these conditions and to perform somatic evaluations. The process must be evaluated based on parameters such as weight, length and cephalic and thoracic perimeters, in order to determine whether weight is adequate for height and whether growth is following the normal pattern.^2^^,^^3^^,^^4^

In the specific case of the initial years of life, weight and length measurements are important indicators of growth.^6^^,^^7^^,^^8^^,^^9^ Growth can be compared with a reference tool such as the curves published by the World Health Organization. Such comparisons can be made periodically, with demonstrations using graphs of postnatal growth patterns.

In Brazil, the National Demography and Health Survey (Pesquisa Nacional sobre Demografia e Saúde, PNDS)^10^ found that for the initial 24 months of life, the length-for-age indicator proved to be the most pronounced measurement of malnutrition. Length deficit was found to affect 7.0% of children, while weight deficit affected 1.7%.

On the other hand, overweight and obesity have shown increasing prevalence because they have been associated with inadequate feeding behavior.^10^ Monte and Giugliani^11^ reported that the complementary feeding must be appropriate for weaning, to enable optimum growth and development. However, in nutritional terms, early introduction of complementary feeding could bring negative consequences, such as the introduction of inappropriate nutrients and decreased duration of breastfeeding, with increased risk of diseases.^9^

Campinas, a city with over one million inhabitants located in the interior of the State of São Paulo, Brazil, that is considered to present high development (human development index, HDI, of 0.85),^12^ has been collecting information on the growth of its children in some regions of the municipality, with distinct samples.

## OBJECTIVE

The objective of the present study was to investigate the weight and length of children less than two years of age, according to the type and duration of breastfeeding, taking into consideration the socioeconomic and demographic characteristics of their families. This was based on a cross-sectional epidemiological survey that aimed to attain an overview of the anthropometric data of infants living in the municipality.

## METHODS

This cross-sectional study was carried out using a random draw in the city of Campinas, State of São Paulo, Brazil. The list of newborns was obtained from the live births registry (Sistema de Informação sobre Nascidos Vivos, SINASC),^13^ which covers 98% of births. This database is composed of roughly 30 variables (including ethnicity, i.e. the color of the child’s skin, as informed by the mother, and the address of the adult responsible for the child), with access on request, accompanied by the appropriate authorization.

Since this study was to be descriptive in nature, the sample was calculated as a proportion of a finite population, using the prevalence of breastfeeding in the municipality (as described in previous years) as the source of information. The month following the birth was used for sampling and a sample error of 0.065% was chosen, thus stipulating a sample size of 2747 infants. Bearing in mind losses and refusals to participate, 3000 infants were drawn and 2857 were included in the study.

Cross-sectional epidemiological studies that are based on interview data can be affected by factors^4^^,^^5^^,^^6^^,^^7^ such as the memory of the interviewed research subjects, use of secondary subjects, subjects’ refusal to participate in the research, inability to locate the potential participants in the study, changes of address, confidentiality of the data informed and the way in which the data obtained is treated. In the present study, 7.3% of the total number of families were not found and 2.5% did not agree to participate. The techniques used to minimize the chances of errors included checking the data using both the birth certificate and SINASC records; making additional draws for the questionnaires; conducting interviews with the mothers by means of the status quo method, which avoids recent events that would affect the mother’s memory; holding the interviews at the University where the research was conducted; making larger draws in relation to the total sample size, in order to reach or exceed the statistically calculated number; and sending out letters containing information about the purpose of this study to public health offices, maternity hospitals and district councils.

Between the months of May 2004 and May 2005, 2857 households with children less than two years of age were visited. The mothers were interviewed at their homes after previously establishing an appointment by telephone. The participants’ physical, moral and social wellbeing was always respected. All of the professionals involved were trained to use the tools developed for the survey. The socioeconomic and demographic information obtained enabled classification of the participants’ social level.^12^^,^^14^^,^^15^ The interviewers weighed and measured these infants between zero and twenty-four months of age, by using portable easy-to-handle equipment, and with due regard to the technical instructions.^7^^,^^16^^,^^17^ Participation in the study was voluntary and was only possible after the adult responsible for the child had filled out the free and informed consent statement. The study was approved by the Research Ethics Committee of the Universidade Estadual de Campinas (Unicamp) (CEP 533/2002).

### Survey and anthropometry

The conditions of the dwellings (type of construction, water supply and sewage system); mother’s profession (university-level; university-level in the healthcare sector, i.e. physician, psychologist, dietician, speech therapist, physiotherapist, occupational therapist, nurse, dentist or pharmacist; non-graduate level employees; or unemployed at the time of the interview; income distribution (expressed as multiples of the minimum monthly salary); and number of household appliances (from zero to four items: refrigerator, freezer, stove, color or black-and-white TV, radio, telephone, cell phone, computer and access to the internet), were used in the study as references for the socioeconomic level and living conditions of the children and their families (IBGE/PNAD).^12^^,^^14^^,^^15^

“Breastfeeding categories”, in accordance with World Health Organization (WHO) recommendations, were used for filling out information on breastfeeding:^2^ exclusive breastfeeding, predominant breastfeeding and full breastfeeding. In exclusive breastfeeding, the infant only drinks milk directly from the breast or milk extracted from it, without any addition of other liquids or solids, with exception of vitamins, minerals or medications in the form of drops or syrups. In predominant breastfeeding, the infant receives breast milk and may additionally receive liquids such as water, tea and fruit juices. In full breastfeeding, maternal milk provision is associated with solid and semi-solid foods, including non-human milk.

The infants’ weights and lengths were measured by the interviewers at their homes, using portable equipment in accordance with the manufacturers’ instructions.^7^^,^^16^^,^^17^ A portable Soehnle pediatric balance was used for weights. A portable Seca anthropometer was used for lengths. Anthropometric data were shown as percentiles and Z-scores, correlating body mass index (BMI) and length-for-age. Cutoff points of under 15 and over 85 for percentiles and between -1 and +1 for Z-scores were selected to classify low weight and short length, and overweight and long length, respectively. The Epi-Info software^18^ and the Statistical Package for the Social Sciences (SPSS) statistical software^19^ were used to make comparisons with the new growth curve recommended by the World Health Organization (WHO 2006; Anthro software).^20^^,^^21^^,^^22^

### Data processing and analysis

In order to describe the sample profile according to the study variables, frequency tables of category variables were constructed containing absolute values (N) and percentages (%). Descriptive statistics on continuous variables were produced using median values. In order to study BMI and length curves, percentiles and Z-scores were calculated for each age group and gender. The binomial test (Z) was used to compare the proportions between percentile values found among children in Campinas and the WHO 2006 reference values. Linear regression analysis was used in order to fit the curves to the original data. The statistical significance level used for all tests was 5% (P < 0.05).

## RESULTS

Table 1 describes the sociodemographic profile of the population evaluated. Boys accounted for 50.7% of the children. Most mothers had studied beyond elementary school and either were working in non-graduate level professions or declared that they were housewives. University-level degrees were held by 6.7%, among which 2.7% related to the healthcare sector. Monthly income associated with the number of household appliances allowed most of the study population to be classified as economic class C, in accordance with the categories of the Brazilian Institute for Geography and Statistics (Instituto Brasileiro de Geografia e Estatística; IBGE),^12^^,^^14^^,^^15^ although all classes were represented.

Table 2 shows that the teenage mothers breastfed less, as did the mothers who were living without a partner and those who were working in technical professions. The mothers who were working in healthcare-related professions breastfed for longer. During the first month of life, 66.2% of the children were on exclusive breastfeeding and 83.1% were on full breastfeeding. Maternal schooling level influenced breastfeeding such that the children whose mothers had had education for either smaller or greater numbers of years received exclusive breastfeeding for longer periods.

Table 3 shows that only the weight curve for the female gender presented a significant effect on the interaction between predominant breastfeeding and maternal schooling. The children whose mothers had had schooling for between 9 and 11 years and who had received predominant breastfeeding for longer than 120 days showed higher weight than the other children did (0.37 ± 0.16; P = 0.020). Even though the rate of predominant breastfeeding was significant in the multiple model (-0.31 ± 0.14; P = 0.027), it was lower among the children who were breastfed for longer period. This result should be taken into account because of the significant interaction.

Table 4 shows the linear regression analysis on weight and length according to the gender. There was a significant difference in the weight curve for male infants according to the mother’s profession. Infants whose mothers did not have a job presented lower weight than did those whose mothers had non-graduate level education or low schooling levels (-0.43 ± 0.16; P = 0.007 for predominant breastfeeding). On the other hand, there was a significant difference in the weight curve relating to predominant breastfeeding among the female children. Infants who received predominant breastfeeding for more than 120 days presented lower weight than did those with shorter breastfeeding periods (-0.18 ± 0.09; P = 0.047). There was also a significant difference in the length curve in relation to the mother’s profession, such that the children whose mothers were in healthcare professions presented shorter length than did those whose mothers had technical education or low schooling levels (-1.61 ± 0.76; P = 0.034 for exclusive breastfeeding).

Table 5 shows that the result from the binomial test for differences between expected (WHO) and observed (Campinas) values was significant for the three BMI percentile ranges analyzed. Lower numbers of children among the sample had eutrophic values and higher numbers presented overweight/obesity, in relation to reference values. Regarding height, all ranges were significant: the highest negative percentage difference was between P15 and P49 (-3.81%) and the highest positive difference was between P85 and P96 (5.2%).

**Figure 1.**
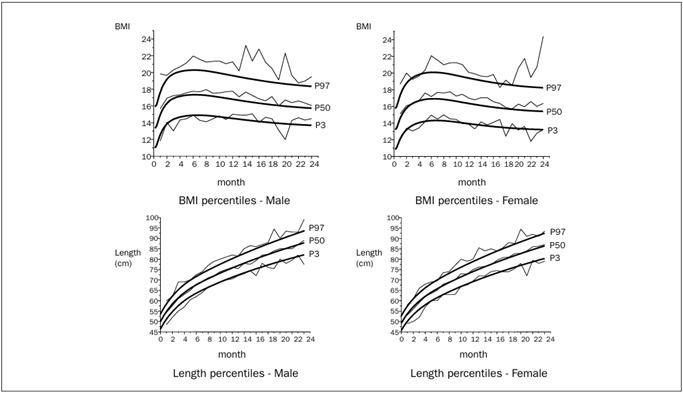
Body mass index (BMI) and length percentiles for male and female children, compared with World Health Organization (WHO 2006) references. Campinas, Brazil, 2004-2005.

**Figure 2.**
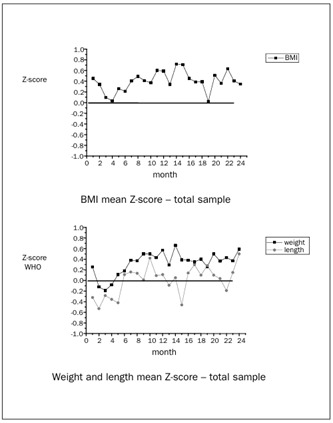
Body mass index (BMI) and weight and length mean Z-scores of total sample, compared with World Health Organization (WHO) 2006 references. Campinas, Brazil, 2004-2005.

In relation to international reference graphs,^20^^,^^21^^,^^22^ 61.25% of the children presented BMI between P15-P85, while 61.12% were within the range for length-for-age; 2.8% were below P3 for BMI and 6.9% for length, while 10.0% were above P97 for BMI and 6.65% for length (Figure 1). The median Z-score was 0.59 (-2.76 to +3.13) for BMI, and 0.11 (-2.42 to +3.69) for length at 12 months of age. The median was 0.34 (-2.13 to +4.98) for BMI and 0.50 (-3.15 to +3.88) for length at 24 months, in both genders (Figure 2).

**Table 1.** Sociodemographic status of study families, Campinas, Brazil, 2004-2005

Variables	Sample (n)	Percentage (%)
Maternal schooling (years)		
≤ 4	222	7.8
5 ├ 8	814	28.5
9 ├ 11	1278	44.7
≥ 12	543	19.0
Maternal occupation		
Housewife	1155	40.4
University level	114	4.0
University level, in healthcare sector^*^	77	2.7
Non-graduate level	1310	45.9
Unemployed	201	7.0
Household income (minimum monthly salaries)		
≤ 2	639	22.4
2 ├ 3	764	26.7
3 ├ 6	729	25.5
6 ├ 10	277	9.7
≥ 10	448	15.7
Total number of household appliances^†^		
≤ 4	560	19.6
5 ├ 9	1660	58.1
10 ├ 14	525	18.4
15 ├ 19	103	3.6
≥ 20	9	0.3

*Maternal occupation (university level, in healthcare sector): physician, psychologist, dietician, speech therapist, physiotherapist, occupational therapist, nurse, dentist or pharmacist; †Total number of household appliances: refrigerator, freezer, stove, color or black-and-white TV, radio, telephone, cell phone, computer and access to the internet.

**Table 2.** Exclusive breastfeeding and full breastfeeding correlated with sociodemographic variables, Campinas, Brazil, 2004-2005

Variables	Median (days)	
	Exclusive breastfeeding (95% CI)	Full breastfeeding (95% CI)
Maternal age (years)		
≤ 20	60 (49.8-70.2)	120 (109.1-130.9)
20 ├ 34	90 (87.2-92.8)	120 (116.5-123.5)
≥ 35	60 (50.9-69.1)	120 (110.7-129.3)
Maternal occupation		
Housewife	90 (85.8-94.2)	120 (113.1-126.9)
University level	120 (94.8-145.2)	120 (100.8-139.2)
University level, in healthcare sector	120 (98.0-142.0)	150 (134.2-165.8)
Non-graduate level	60 (56.6-63.4)	120 (116.1-123.9)
Unemployed	60 (46.8-73.2)	120 (110.1-129.9)
Maternal schooling (years)		
≤ 4	90 (76.7-103.3)	120 (110.0-130.1)
5 ├ 8	60 (55.5-64.5)	120 (113.0-127.0)
9 ├ 11	60 (56.3-63.7)	120 (115.5-124.5)
≥ 12	90 (80.0-100.0)	120 (113.4-126.6)
Marital status		
Unmarried	60 (56.6-63.4)	120 (112.5-127.5)
Married	90 (87.2-92.8)	120 (116.5-123.5)

**Table 3.** Linear regression analysis on weight and length according to gender, age, breastfeeding (< P50 versus ≥ P50) and maternal schooling, in terms of the interaction between breastfeeding and maternal schooling, expressed as slope, standard error and P-value. Campinas, Brazil, 2004-2005

Dependent variable	Independent variable	Slope	Standard error	P-value
Weight (male)	Intercept	4.46	0.12	< 0.001
	Age	0.67	0.02	< 0.001
	Age (squared term )	-0.014	0.001	< 0.001
	Breastfeeding (≥ P50)	-0.036	0.133	0.785
	Maternal Schooling 1^*^	-0.064	0.175	0.715
	Maternal Schooling 2	0.058	0.118	0.624
	Maternal Schooling 3	0.012	0.110	0.915
	IB versus MS 1^†^	-0.148	0.251	0.557
	IB versus MS 2	-0.122	0.175	0.486
	IB versus MS 3	0.011	0.158	0.944
Length (male)	Intercept	3.61	0.34	< 0.001
	Age	2.45	0.05	< 0.001
	Age (squared term )	-0.045	0.002	< 0.001
	Breastfeeding (≥ P50)	0.245	0.377	0.517
	Maternal Schooling 1^*^	-0.591	0.498	0.235
	Maternal Schooling 2	-0.055	0.334	0.870
	Maternal Schooling 3	0.048	0.312	0.877
	IB versus MS 1^†^	-0.630	0.713	0.377
	IB versus MS 2	-0.019	0.497	0.970
	IB versus MS 3	-0.259	0.447	0.562
Weight (female)	Intercept	4.05	0.12	< 0.001
	Age	0.64	0.02	< 0.001
	Age (squared term)	-0.013	0.001	< 0.001
	Breastfeeding (≥ P50)	-0.305	0.138	0.027
	Maternal Schooling 1^*^	0.011	0.155	0.942
	Maternal Schooling 2	-0.054	0.114	0.636
	Maternal Schooling 3	-0.122	0.109	0.263
	IB versus MS 1^†^	-0.075	0.251	0.765
	IB versus MS 2	0.071	0.172	0.678
	IB versus MS 3	0.373	0.161	0.020
Length (female)	Intercept	51.58	0.32	< 0.001
	Age	2.54	0.05	< 0.001
	Age (squared term)	-0.046	0.002	< 0.001
	Breastfeeding (≥ P50)	-0.370	0.383	0.334
	Maternal Schooling 1^*^	0.041	0.430	0.925
	Maternal Schooling 2	0.069	0.317	0.828
	Maternal Schooling 3	-0.183	0.303	0.545
	IB versus MS 1^†^	-0.619	0.697	0.375
	IB versus MS 2	-0.265	0.477	0.579
	IB versus MS 3	0.561	0.447	0.209

*MS = maternal schooling period; MS 1 = less than 4 years, MS 2 = between 5 and 8 years, MS 3 = between 9 and 11 years, IB = interaction breastfeeding, †IB versus MS = interaction between breastfeeding and maternal schooling.

**Table 4.** Linear regression analysis on weight and length according to age, sex, breastfeeding (< P50 versus ≥ P50) and maternal occupation, in terms of the interaction between breastfeeding and maternal occupation, expressed as slope, standard error and P-value. Campinas, Brazil, 2004-2005

Dependent variable	Independent variable	Slope	Standard error	P-value
Weight (male)	Intercept	4.51	0.10	< 0.001
	Age	0.67	0.02	< 0.001
	Age (squared term )	-0.014	0.001	< 0.001
	Breastfeeding (≥ P50)	-0.114	0.091	0.209
	Maternal Occupation 1^*^	-0.061	0.084	0.466
	Maternal Occupation 2	0.080	0.221	0.718
	Maternal Occupation 3	-0.212	0.314	0.500
	Maternal Occupation 4	-0.425	0.158	0.007
	IB versus MO 1^†^	0.032	0.128	0.804
	IB versus MO 2	0.077	0.299	0.798
	IB versus MO 3	0.017	0.381	0.965
	IB versus MO 4	0.358	0.236	0.129
Length (male)	Intercept	53.61	0.27	< 0.001
	Age	2.46	0.05	< 0.001
	Age (squared term )	-0.04	0.02	< 0.001
	Breastfeeding (≥ P50)	0.053	0.258	0.836
	Maternal Occupation 1^*^	-0.058	0.238	0.806
	Maternal Occupation 2	0.575	0.627	0.359
	Maternal Occupation 3	-0.443	0.890	0.619
	Maternal Occupation 4	-0.615	0.448	0.170
	IB versus MO 1^†^	-0.071	0.362	0.845
	IB versus MO 2	0.258	0.850	0.761
	IB versus MO 3	0.157	1.082	0.884
	IB versus MO 4	0.198	0.669	0.768
Weight (female)	Intercept	4.05	0.09	< 0.001
	Age	0.64	0.02	< 0.001
	Age (squared term)	-0.013	0.001	< 0.001
	Breastfeeding (≥ P50)	-0.177	0.089	0.047
	Maternal Occupation 1^*^	-0.119	0.080	0.136
	Maternal Occupation 2	0.024	0.214	0.911
	Maternal Occupation 3	-0.221	0.241	0.359
	Maternal Occupation 4	-0.253	0.166	0.127
	IB versus MO 1^†^	0.133	0.125	0.291
	IB versus MO 2	-0.277	0.307	0.368
	IB versus MO 3	0.240	0.381	0.528
	IB versus MO 4	0.187	0.234	0.425
Length (female)	Intercept	51.59	0.25	< 0.001
	Age	2.54	0.05	< 0.001
	Age (squared term )	-0.046	0.002	< 0.001
	Breastfeeding (≥ P50)	-0.089	0.248	0.719
	Maternal Occupation 1^*^	-0.237	0.222	0.284
	Maternal Occupation 2	0.838	0.593	0.158
	Maternal Occupation 3	-0.616	0.669	0.357
	Maternal Occupation 4	0.146	0.461	0.751
	IB versus MO 1^†^	-0.124	0.348	0.722
	IB versus MO 2	-1.332	0.853	0.119
	IB versus MO 3	-0.654	1.057	0.536
	IB versus MO 4	-0.574	0.651	0.378

*MO = maternal occupation; MO 1 = housewife/no job; MO 2 = university degree; MO 3 = university level, in healthcare sector; MO 4 = no job. Professions that require a technical level were used as a reference for comparisons; IB = interaction breastfeeding; †IB versus MO = interaction between breastfeeding and maternal occupation.

**Table 5.** Comparative analysis on body mass index (BMI) and length percentiles between the sample from Campinas, Brazil (2004 - 2005) and the World Health Organization (WHO) 2006 references

Variable	Percentiles	% WHO 2006^*^	% Campinas	Difference (%) between sample and reference	Binomial test^†^
BMI	< P3	3.00	2.80	-0.20	Z = -0.63; P = 0.531
	P3-P14	8.00	8.19	0.19	Z = 0.38; P = 0.708
	P15-P49	35.00	24.78	-10.22	Z = -11.45; P < 0.001
	P50-P84	35.00	36.47	1.47	Z = 1.65; P = 0.099
	P85-P96	8.00	17.71	9.71	Z = 19.13; P < 0.001
	≥ P97	3.00	10.05	7.05	Z = 22.08; P < 0.001
Length	< P3	3.00	6.90	3.90	Z = 12.21; P < 0.001
	P3-P14	8.00	12.15	4.15	Z = 8.17; P < 0.001
	P15-P49	35.00	31.19	-3.81	Z = -4.27; P < 0.001
	P50-P84	35.00	29.93	-5.07	Z = -5.69; P < 0.001
	P85-P96	8.00	13.20	5.20	Z = 10.24; P < 0.001
	≥ P97	3.00	6.65	3.65	Z = 11.44; P < 0.001

*Expected percentage in the respective percentile zone according to WHO 2006 references. †Z-statistic and P-value in relation to the Binomial Test for proportions, comparing the value shown in Campinas sample with the expected values from WHO 2006 references.

## DISCUSSION

The Campinas study was cross-sectional and population-based. It found that weaning occurred early among the children evaluated, given 50% had already been weaned at three months of age. Moreover, the anthropometric status stood out: growing levels of obesity beginning very early among the children under two years of age living in the city, such that 10% of them presented BMI above percentile 97. Multivariate logistic regression models were constructed for this study to identify weight and length changes, and prevalences of 27.76% for excess weight and 19.85% for excess length were observed. This scenario was above the expected values, in comparison with the WHO 2006 reference population (11.0%), thus indicating possible obesity among children under two years of age. The median Z-score was more uniform up to 12 months of age, given that a larger number of children were evaluated (2116), thereby giving higher statistical power to the analysis, despite the variability. Moreover, the standard deviation in relation to the median was found to vary greatly in some cases, as shown in Tables 3 and 4.

The children presented a trend toward changes in nutritional status patterns that may have begun in early childhood.^23^^,^^24^^,,^^25^ In the present study, infants were assessed cross-sectionally by means of a random sample and a representative number of children for the population of this age group in the city. This was useful for diagnosing the nutritional status of infants in the city. However, the fact that cross-sectional studies generally find associations but do not establish a cause-effect relationship between the variables should be taken into account. BMI values above reference values were found mainly among children on full breastfeeding.

Based on multiple regression analysis, a significant correlation with maternal variables was found. Boys whose mothers were unemployed had lower weights than those born from employed mothers. Regarding length, children whose mothers worked as health professionals were the shortest, although this result may have been random or may have shown features of this specific population. The National Demography and Health Survey (PNDS, 2006)^10^ showed an association between mothers’ schooling and malnutrition. Mothers with no schooling or who had attended school for only one to three years were 11 times more likely to have malnourished children than were mothers with 12 or more years of schooling. In Campinas, 44.5% of mothers had had between 9 and 11 years of education and families living with an income of not more than six minimum monthly salaries were uniformly distributed, although 15.7% were living with an income of over ten minimum monthly salaries. Social indicators associated with the median levels of exclusive or full breastfeeding may have contributed to the nutritional status found, which differed from the national survey, thus showing that initial obesity was occurring at an early age.

Longo et al.^26^ conducted a study in all of the five Brazilian geographical regions on more than 3000 infants. They concluded that there was a positive association between the speeds of length and weight gain during exclusive breastfeeding and full breastfeeding and the maternal schooling level. Moreover, they concluded that the type of breastfeeding had a positive influence on the adequacy of growth. The data obtained from our study are in agreement with their study, such that maternal schooling levels and professions were associated with the duration of the different types of breastfeeding and with growth.

Breastfeeding duration and growth were the focus of another study^27^ conducted in Belém (Pará, Brazil) that reported the effects of breastfeeding practices on growth rates, particularly between the sixth and seventh months of age. In the present study, the type of maternal breastfeeding mainly affected the length of the infants, when associated with the mother’s profession.

The initial assumptions were that children under two years of age would tend to become malnourished when not breastfed, as would those living under less privileged social conditions. The relationship between length deficits among infants and maternal schooling levels among mothers in healthcare professions is unclear. This was particularly so because the assumption was that the children whose mothers worked in healthcare professions would be less malnourished because these women would have more information on diet and breastfeeding techniques. Thus, this finding may have been random, given that less than 3% of the infants’ mothers worked in healthcare professions.

The results from three household survey studies conducted in the city of São Paulo, Brazil, in 1974-1975, 1984-1985 and 1995-1996,^6^^,^^28^^,^^29^^,^^30^ had already shown developments within growth patterns, in which although malnutrition had been brought under control, it had not yet been solved. Thus, obesity began to appear, mainly among the children of wealthier families. The data from the present study demonstrate this trend, even though it was conducted ten years after the last household survey carried out in the State of São Paulo. The deficits in the weight/age and height/age ratios were under 10%, in relation to breast milk feeding types and socioeconomic strata, thus showing that the children were possibly benefiting from adequate diet and health conditions and were growing similarly to the reference population.

The Family Budget Survey (Pesquisa de Orçamentos Familiares, POF, 2004),^31^ which was carried out by the IBGE between 2002 and 2003, showed that the median weight and length for two-year-old children were 12.1 kg and 12.6 kg for weight and 87.7 cm and 88.9 cm for length, for girls and boys respectively. The results for the population of Campinas were 12.24 kg and 87 cm for girls, and 13.00 kg and 89 cm for boys. This shows that the infants living in this municipality are growing similarly to what is found for the whole country.

## CONCLUSIONS

Factors such as the availability of data and surveys, and the number of children evaluated, made it possible to find out about the anthropometric situation, duration of breastfeeding and social conditions of young infants living in Campinas, São Paulo, Brazil. Modernization of the society and increased maternal schooling levels and income levels were positive factors for children’s nutritional status. The maternal schooling and breastfeeding levels probably made the largest contributions towards this situation, which despite being far from the recommendations, is better than the Brazilian average. The data confirmed that this country is undergoing a nutritional transition, with decreasing undernutrition. On the other hand, obesity was found to be occurring early, at the beginning of childhood, among the infants living in Campinas. The study showed that the BMI of a notable proportion of the infants was above reference percentile 97, thus illustrating the trend toward obesity. However, in general, these infants presented a satisfactory nutritional status in comparison with infants living in other places in Brazil.
